# HCO_3_^−^-independent pH Regulation in Astrocytes *in Situ* Is Dominated by V-ATPase* ^♦^

**DOI:** 10.1074/jbc.M115.636597

**Published:** 2015-02-09

**Authors:** Daniel Bloch Hansen, Nestor Garrido-Comas, Mike Salter, Robert Fern

**Affiliations:** From the ‡ Plymouth University Peninsula Schools of Medicine and Dentistry, Plymouth PL6 8BU,; the § Department of Cell Physiology and Pharmacology, University of Leicester, Leicester LE1 7RH, and; the ¶ Institute of Membrane and Systems Biology, University of Leeds, Leeds LS2 9JT, United Kingdom

**Keywords:** Astrocyte, pH Regulation, Proton Transport, Sodium-Proton Exchange, Vacuolar ATPase, Glia, Acid Extrusion, Optic Nerve, White Matter

## Abstract

The mechanisms of HCO_3_^−^-independent intracellular pH (pH*_i_*) regulation were examined in fibrous astrocytes within isolated neonatal rat optic nerve (RON) and in cultured cortical astrocytes. In agreement with previous studies, resting pH*_i_* in cultured astrocytes was 6.82 ± 0.06 and inhibition of the V-ATPase H^+^ pump by Cl^−^ removal or via the selective inhibitor bafilomycin had only a small effect upon resting pH*_i_* and recovery following an acid load. In contrast, resting pH*_i_* in RON astrocytes was 7.10 ± 0.04, significantly less acidic than that in cultured cells (*p* < 0.001), and responded to inhibition of V-ATPase with profound acidification to the 6.3–6.5 range. Fluorescent immuno-staining and immuno-gold labeling confirmed the presence V-ATPase in the cell membrane of RON astrocyte processes and somata. Using ammonia pulse recovery, pH*_i_* recovery in RON astrocyte was achieved largely via V-ATPase with sodium-proton exchange (NHE) playing a minor role. The findings indicate that astrocytes in a whole-mount preparation such as the optic nerve rely to a greater degree upon V-ATPase for HCO_3_^−^-independent pH*_i_* regulation than do cultured astrocytes, with important functional consequences for the regulation of pH in the CNS.

## Teaser

**Background:** Astrocyte pH regulation is essential for brain function.

**Results:** Bicarbonate-independent acid load recovery in whole-mount astrocytes was achieved primarily via V-ATPase, which also held astrocytes at a basic resting pH.

**Conclusion:** V-ATPase is the major bicarbonate-independent H^+^ extruder in a whole-mount astrocyte population.

**Significance:** High levels of V-ATPase expression will isolate astrocyte pH regulation from cytoplasm changes in other ion species.

## Introduction

Astrocytes are responsible for extracellular ion homeostasis in the CNS, including H^+^. pH can have profound effects upon excitability, neurotransmission, and injury responses, and the mechanisms of pH regulation in astrocytes have been examined in detail previously. Acute intracellular pH (pH*_i_*) balance in the CNS is determined by two factors: (i) the intrinsic buffering power of neural cells, and (ii) the activity of membrane transporters that move H^+^ equivalents across cell membranes, resulting in cellular acid extrusion or acid loading (1–3). Acid extruders can either directly remove H^+^ from cells or import HCO_3_^−^, whereas acid loaders are thought to exclusively remove HCO_3_^−^. Two HCO_3_^−^-dependent transporters, the Na-HCO_3_ cotransporter and the Na^+^-driven Cl-HCO_3_ exchanger, have been described in astrocytes (3, 4) in addition to an electro-neutral Cl-HCO_3_ exchanger (5). HCO_3_^−^-independent transporters maintain intracellular pH by transporting H^+^ either directly or coupled to another ionic gradient. Studies using cultured astrocytes indicate that the Na-H exchanger (NHE)^2^ is the principal HCO_3_^−^-independent H^+^ extrusion protein in the CNS, coupling H^+^ extrusion to the inward Na^+^ gradient (3, 6–9). Astrocytes also express a V-type H-ATPase that can be blocked by bafilomycin, although the involvement of this pump in pH maintenance and recovery following an acid load is limited in cultured cells (10, 11).

The majority of information available regarding H^+^ extrusion from astrocytes was obtained from culture preparations. These have the advantage of low background signal and ease of use but the disadvantage that the cells may behave differently from cells *in situ*, where they are integrated into complex neural networks. Using postnatal day 0–4 (P0–P4) rat optic nerves, we have now examined the involvement of V-ATPase and NHE in the maintenance of steady-state pH*_i_* and pH*_i_* recovery following an acid load in astrocytes. This white matter tract is pre-myelinated, allowing high-contrast imaging of ion-sensitive intracellular dyes, and contains a population of early maturing fibrous astrocytes. The nerve was transected at either end, and cells were imaged in the central region and are therefore in a native, effectively undamaged environment. We report that in contrast to cultured astrocytes, V-ATPase is the dominant HCO_3_^−^-independent H^+^ extrusion mechanism in this *in situ* astrocyte population.

## EXPERIMENTAL PROCEDURES

### 

#### 

##### Rat Optic Nerve Preparation

Postnatal day 0–2 (P0–P2) Lister Hooded or P0–P4 Wistar rats of both sexes were humanely killed, and the optic nerves were dissected according to regulations from the Home Office, United Kingdom, with approval from the local Animal Welfare and Ethical Review Board (AWERB) of the University of Leicester and Plymouth University. The nerves were placed in HEPES-buffered artificial cerebrospinal fluid (aCSF) (see below). The pH-sensitive fluorescent dye 2′,7′-bis-(2-carboxyethyl)-5 (and 6)-carboxyfluorescein (BCECF) (Molecular Probes Inc.) was used to measure pH*_i_*. BCECF-AM (10 μm) was dissolved in dimethyl sulfoxide, 10% Pluronic acid, and cells were loaded in HEPES-aCSF at room temperature for 90 min (15 min for cultures). After washing, the ends of the nerves were fixed to a glass coverslip with a small amount of cyanoacrylate glue and sealed into a Plexiglas perfusion chamber with silicone grease (atmosphere chamber, Warner Instruments). The chamber was mounted on an Eclipse TE2000-U inverted microscope (Nikon), continuously superfused at a rate of 2 ml/min, and maintained in an oxygenated environment (see Ref. 12) for further details). The chamber temperature was maintained at 37 °C with a flow-through feedback tubing heater (Warner Instruments) positioned immediately before the chamber and a feedback objective heater (Bioptechs) that warmed the objective to 37 °C. This combination of heating systems regulated the temperature of the bath and coverslip to 37 °C, as established periodically with a temperature probe.

##### Astrocyte Cultures

Astrocytes were isolated and cultured using an established protocol (13). Brains from BALB/c mice embryos (embryonic days 14–16) were collected in Hanks' balanced salt solution (Gibco) on ice, and after removal of the meninges, the tissue was digested for 2 min at 37 °C with 2 ml of 1% trypsin solution. The digestion was stopped with 10 ml of DMEM (Gibco) with 10% FCS (Gibco). After washing, the tissue was triturated in 2 ml of 0.5% DNase solution. 10 ml of DMEM, 10% FCS medium was added, and cells were centrifuged for 5 min at 200 × *g*. The pellet was resuspended in DMEM, 10% FCS incubation media with 50 units/ml penicillin and 50 mg/ml streptomycin (P/S) (Invitrogen), and the cells were plated in poly-l-lysine (100 μg/ml) precoated flasks (1.8–3.5 × 10^7^ cells/flask). The medium was partially exchanged every 3 days. After 15–20 days, a mixed glial culture was achieved. The cells were separated by shaking, leaving the astrocyte layer, which was washed twice with serum-free Hanks' balanced salt solution prior to trypsinization. Glial fibrillary acidic protein (GFAP) staining of sister cultures showed 93.9% of cells being GFAP^+^. Astrocytes were centrifuged at 4 °C for 5 min at 200 × *g* and resuspended with incubation media DMEM, 10% FCS, P/S and plated on coverslips, which were incubated at 37 °C until mounted in an atmosphere chamber as described above.

##### Cell Imaging

**FIGURE 1.**
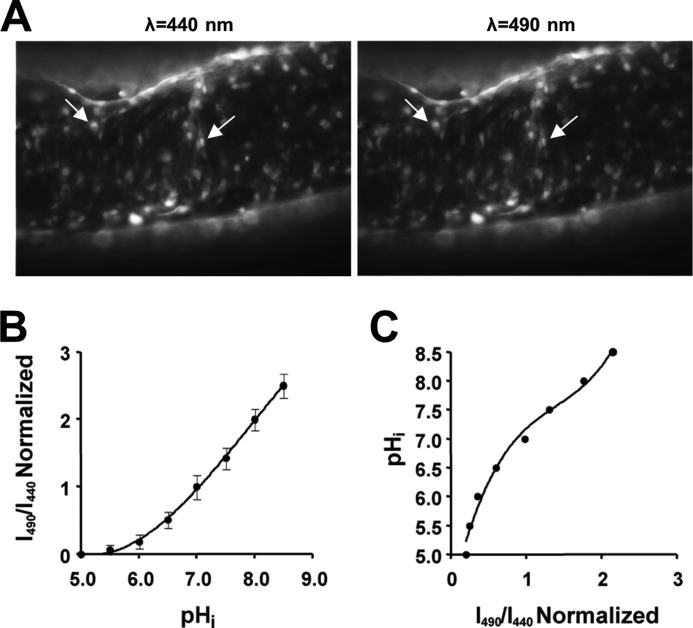
**Cell loading of BCECF-AM and pH*_i_* calibration in optic nerve astrocytes.** *A*, images of BCECF-AM loading in rat optic nerve astrocytes. A large number of cells can be seen to have loaded the dye (*e.g. arrows*) during excitation at 440 and 490 nm. *B*, plot of the calibration curve obtained from optic nerve astrocytes. 490/440 ratios were obtained after exposing cells to high K^+^/nigericin calibration solutions at pH values from 5.5 to 8.5. The 490/440 ratio was normalized to the ratio at pH 7.0. Values are given as mean ± S.E. *C*, replotted calibration curve with the data fitted to a third order polynomial function. This is used for correlating pH*_i_* values to normalized 490/440 ratios obtained from single-point calibrations at the end of experiments.

Cells within the optic nerve or on coverslips were illuminated at 440 and 490 nm by a monochromator (Optoscan; Cairn Research), and images were collected every 30 s at 510 nm using an appropriate filter set (Chroma Technology) and a cooled CCD camera (CoolSNAP HQ; Roper Scientific). Cells were initially brought into focus during illumination at 490 nm, usually selecting a field containing >30 cells in optic nerves and >60 in astrocyte cultures. In the case of optic nerves, all the cells loading the dye at this age are astrocytes (12), and a typical optic nerve cell loading with BCECF is shown in Fig. 1*A*. The brightest 15 or 30 cells were selected for analysis to avoid data biasing between nerves or cultures, respectively. Once mounted in the microscope, nerves or cell cultures were left to equilibrate for 10–20 min in HEPES-aCSF to remove any unhydrolyzed dye before the start of each experiment. Regions of interest were drawn around individual cells, and following background subtraction, the fluorescence ratio for each was plotted against time.

##### Calibration

The 490/440 fluorescence ratio was calibrated against a lookup table giving a single point reference at the end of each experiment. The lookup table was produced by superfusing a series of optic nerves with a high K^+^/nigericin solution driving pH*_i_* to that of the superfusate and giving one point on a 490/440 *versus* pH*_i_* plot (8, 14). The high K^+^/nigericin solution contained (in mm): 10 Na^+^, 130 K^+^, 10 Cl^−^, 130 gluconic acid, 0.6 Ca^2+^, 0.6 MgSO_4_, 10 glucose, and 10 PIPES for pH ≤ 6.8 or 10 HEPES for pH ≥ 7.0. For Nigericin (10 μm), gramicidin (5 μm), and ouabain (1 mm) solutions, pH was adjusted with NaOH, giving eight different solutions with pH ranging from 5.0 to 8.5 in intervals of 0.5. 490/440 ratios were calculated to obtain the calibration curve shown in Fig. 1*B*, normalized to pH 7.0. As in previous studies (8, 14), the calibration curve was replotted and fitted with a third order polynomial function obtaining a best-fit value *R*^2^ of 0.98 (Fig. 1*C*). The 490/440 data from individual experiments was then converted to pH*_i_* values by exposing the cell to a pH 7.0 calibration solution at the end of each experiment, applying the resulting 490/440 value to the third order polynomial function and converting the whole data series using these values.

##### Acid Loading and H^+^ Buffering

Rat optic nerves or astrocyte cultures were acid-loaded using the NH_4_^+^ prepulse technique (15). Briefly, nerves or cultures were prepared as described above and exposed to HEPES-aCSF containing 20 mm (NH_4_)_2_SO_4_. NH_3_ freely permeates the cell membrane and reacts with free H^+^ in the cytoplasm, causing a rapid increase in pH*_i_*. Slow NH_4_^+^ influx follows, acidifying the cell, and subsequent removal of the extracellular NH_3_/NH_4_^+^ causes a rapid decrease in pH*_i_* to below the starting value. Optic nerve astrocytes were exposed to NH_3_/NH_4_^+^ for 5 min, whereas exposure time for cultured astrocytes was reduced to 2 min due to problems with cell viability. The shorter exposure time for cultured astrocytes allowed for a full recovery of pH*_i_* consistent with Ref. 1. The shorter ammonia pulse also caused a smaller acidification and thus recovery over a narrowed band of pH*_i_* values (6.6–7.0) as compared with optic nerve astrocytes.

H^+^-buffering power was calculated using an established protocol (1, 8, 14). The total cellular H^+^-buffering capacity (β**_T_**) is the product of an HCO_3_-dependent component (β_HCO3_ = 2.3 [HCO_3_^−^]*_i_*) and an intrinsic component (β_i_). By measuring the pH*_i_* increases produced by exposing cells to different external concentrations of NH_4_^+^ under conditions where pH*_i_* regulation is interrupted (8) and in the absence of HCO_3_^−^, β**_T_** = β_i_, which can be calculated as

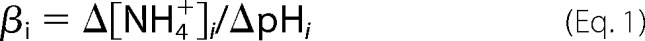


Nerves were initially superfused with HEPES-aCSF followed by HEPES-aCSF with Na^+^ and Cl^−^ substituted to block pH regulation in the astrocytes (see below). Three rat optic nerves (RONs) were exposed to [NH_4_^+^] in concentrations of 1, 2.5, 5, 10, or 20 mm. Following each pulse, pH*_i_*, was extrapolated to time 0, Δ[NH_4_^+^]*_i_* was calculated using the Henderson-Hasselbalch equation, and β**_T_** = β_i_ was calculated and plotted against the resulting pH*_i_* (4, 8).

##### Rat Optic Nerve Extracellular pH

Ion-sensitive microelectrodes were manufactured as described previously (16). Electrodes were pulled from glass capillaries (1.5-mm outer diameter, 0.86-mm inner diameter, Harvard Apparatus) with a Sutter model P-97 puller (Sutter Instrument Co.). Electrodes were salinized using *N*,*N*-dimethyltrimethylsilylamine (TMSDMA), backfilled with HEPES-aCSF (pH 7.45), and front-filled with the ion-exchange resin (hydrogen ionophore I: mixture A, Sigma-Aldrich). The reference electrode was pulled as above and filled with HEPES-aCSF. Experimental solutions were kept at 37 °C and continuously aerated. The Ag-AgCl bath electrode was connected to the superfusate using a 1 m KCl agar bridge to avoid drift in the Cl^−^-free solution. Dissected rat optic nerves were stored in HEPES-aCSF for at least 1 h to allow axon sealing. Nerves were then fixed onto coverslips as described above. Following impalement, the nerve was left to recover for 5–10 min before the start of experiments. The recordings were performed and recorded with a high impedance differential electrometer (FD223a, World Precision Instruments), a IX-228 data acquisition unit, and LabScribe2 software (both iWorx Systems, Inc.) with sampling at 1 Hz.

Due to differences in composition between electrode fillings and the experimental Na^+^/Cl^−^-free HEPES-aCSF, we compensated for the junction potential by recording the change in junction potential in the chamber following experiments and subtracting it from the in-nerve recordings. The electrodes were calibrated using HEPES-aCSF with pH levels ± 1 unit relative to the control (6.4 and 8.4, respectively). pH sensitivity of the electrodes was found to be similar in control and Na^+^/Cl^−^-free (NMDG gluconate-substituted) HEPES-aCSF.

##### Antibody Staining

Nerves were transferred to 0.1 m PBS prior to fixing in 4% paraformaldehyde for 30 min at room temperature. The nerves were then cryoprotected (20–30% sucrose for 5 min), transferred to Tissue-Tek medium (Sigma), and frozen using ethanol and dry ice. 20-μm sections were cut by cryostat and mounted onto microscope slides. The freshly cut sections were then submerged in 0.1 m PBS for 5 min and blocked in 0.1 m PBS containing 10% goat serum and 0.5% Triton-X for 120 min at room temperature prior to overnight exposure to primary antibody at 4 °C in the same solution. Monoclonal GFAP (Molecular Probes, 1:200) and rabbit anti-V-ATPase F (sc-20947, Santa Cruz Biotechnology, 1:100) were used. Slides were then washed (3 × 5 min) and incubated in the appropriate Alexa Fluor-conjugated secondary antibody (Molecular Probes, 1:1000) for 60 min at room temperature. The sections were then washed sequentially in 0.5, 0.1, and 0.05 m PBS containing 10% normal serum. Images were collected using an Olympus IX70 confocal microscope (60× objective). In all cases, controls treated as above but without primary antibody were blank.

##### Electron Microscopy

Optic nerves were post-fixed in 3% glutaraldehyde/Sorenson's Ringer prior to post-fixing with 2% osmium tetroxide and dehydration prior to infiltration in epoxy. Sections were counterstained with uranyl acetate and lead citrate and examined with a Jeol 100CX electron microscope (see Ref. 17 for details). For post-embedding immuno-labeling, primary antibody was applied to the sections overnight, and appropriate 20-nm gold particle secondary antibodies were applied following washing (see Ref. 18 for further details).

##### Solutions and Chemicals

HEPES-buffered aCSF contained (in mm): 126 NaCl, 4 KCl, 2 NaH_2_PO_4_, 2 MgSO_4_, 25 HEPES, 10 glucose, 2 calcium gluconate, 2 sodium cyclamate, pH 7.45, adjusted with HCl. For Na^+^-free HEPES-buffered aCSF, NaCl was replaced by 128 mm NMDG Cl^−^ and 2 mm KCl was replaced by 2 mm KH_2_PO_4_, omitting both sodium cyclamate and NaH_2_PO_4_. For Cl^−^-free aCSF, NaCl was replaced by 128 mm sodium cyclamate, and KCl was replaced by 2 mm K^+^ gluconate and 2 mm KH_2_PO_4_. The Na^+^- and Cl^−^-free solution contained (in mm) 2 MgSO_4_, 25 HEPES, 10 glucose, 2 calcium gluconate, 2 potassium gluconate, 2 KH_2_PO_4_, and 130 NMDG gluconate prepared by titrating the NMDG-containing solution with gluconate to pH 7.45. Osmolarity for all solutions was measured using a vapor pressure osmometer (Camlab) and adjusted with sucrose to 315 mosm. All chemicals were purchased from Sigma-Aldrich) unless otherwise stated.

##### Statistics

Static significance was tested using one-way analysis of variance with Bonferroni's post hoc test or Student's *t* test as appropriate (GraphPad, Prism).

## RESULTS

### 

#### 

##### Na^+^- and Cl^−^-dependent pH_i_ Regulation Differs between in Situ and Cultured Astrocytes

**FIGURE 2.**
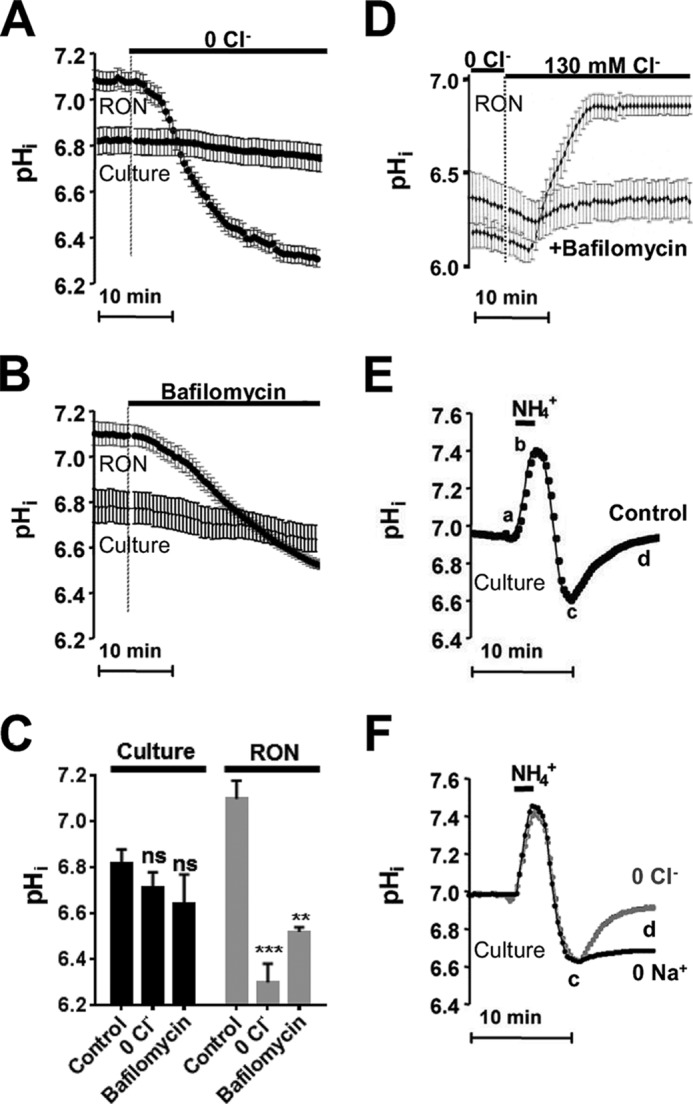
**The effect of bafilomycin or ionic substitution upon HCO_3_^−^-independent pH*_i_* in rat optic nerve and cultured astrocytes.** *A*, substitution of Cl^−^ (0 Cl^−^) in HEPES-buffered, nominally HCO_3_^−^-free, aCSF evokes a large acidification in RON astrocytes, but not in cultured astrocytes (*Culture*). *B*, exposure to the V-ATP blocker bafilomycin (50 nm) evokes a large acidification in optic nerve astrocytes but not in cultured astrocytes. *C*, summary of data in *A* and *B*. Values are given as mean ± S.E. Significance is given relative to the respective control: **, *p* < 0.001, ***, *p* < 0.0001. *ns*, not significant. *D*, pH*_i_* of optic nerve astrocytes acidified by Cl^−^ removal is normalized following restoration of physiological extracellular Cl^−^ in a bafilomycin-sensitive fashion. *E*, recovery of pH*_i_* from an acid load in cultured astrocytes in control solution. Baseline pH*_i_* was stable prior to the onset of a 2 min 20 mm ammonia pulse. *Segment a-b* is the alkalinization evoked by NH_4_ exposure, *segment b-c* is the acid load generated after switching back to ammonia-free conditions, and *segment c-d* is the pH*_i_* recovery. *F*, as in E; pH*_i_* recovery in cultured astrocytes was reduced in the absence of extracellular Na^+^ (0 Na^+^) but unaffected by Cl^−^ substitution (0 Cl^−^).

We determined the contribution of the Cl^−^-dependent V-ATPase to resting pH*_i_* levels in both cultured astrocytes and *in situ* rat optic nerve astrocytes. Optic nerve and cultured astrocytes superfused for 15–25 min with HEPES-aCSF displayed baseline pH*_i_* of 7.10 ± 0.04 (*n* = 4 optic nerves, 60 cells) and 6.82 ± 0.06 (*n* = 4 cultures, 90 cells), respectively (*p* < 0.01; Fig. 2, *A* and *C*). Cl^−^ removal did not significantly affect the pH*_i_* of cultured astrocytes, which fell to 6.71 ± 0.07 (*p* > 0.05; *n* = 60; Fig. 2, *A* and *C*), whereas pH*_i_* in optic nerve astrocytes fell significantly to 6.30 ± 0.04 (*p* < 0.001; *n* = 4 optic nerves, 60 cells; Fig. 2, *A* and *C*). Perfusion with the selective V-ATPase inhibitor bafilomycin (50 nm) reduced optic nerve astrocyte resting pH*_i_* to 6.52 ± 0.02 (*p* < 0.01;c Fig. 2, *B* and *C*) over a similar 20–30-min time course to the acidification produced by Cl^−^ removal. Bafilomycin reduced pH*_i_* in cultured astrocytes by ∼0.15 pH units to 6.64 ± 0.04 (*n* = 4 cultures, 90 cells), which was significantly less than the ∼0.61 pH unit change found in optic nerve astrocytes. The plateau pH*_i_* levels in optic nerve and cultured astrocytes exposed to bafilomycin were not significantly different (*p* > 0.5), and V-ATPase activity in optic nerve cells therefore accounts for their alkaline resting pH*_i_* as compared with cultured cells. To confirm the Cl^−^-dependent nature of the V-ATPase activity in optic nerve astrocyte, pH*_i_* recovery following a 30-min period of 0 Cl^−^ perfusion was shown to be blocked by bafilomycin upon Cl^−^ restoration (Fig. 2*D*).

The differential effect of V-ATPase inhibition between cultured and *in situ* astrocytes suggests higher rates of H^+^ extrusion via this route in optic nerve astrocytes. Following an acid load (exposure to a 20 mm ammonia pulse) in cultured astrocytes, pH*_i_* fell from a baseline of 6.98 ± 0.05 to 6.60 ± 0.07 before recovering over a 7-min period (Fig. 2*E*, *segment c-d*) to 6.96 ± 0.07 (*p* > 0.05 *versus* baseline; *n* = 4 cultures, 90 cells). Na^+^ removal largely abolished the pH*_i_* recovery following an acid load, with pH*_i_* reaching 6.69 ± 0.07 (*n* = 4 cultures, 90 cells; Fig. 2*F*), which is consistent with prior studies that report largely Na^+^-dependent forms of HCO_3_^−^-independent acid extrusion in these cells (8, 9). In contrast, removal of Cl^−^ from the medium had no significant effect upon pH*_i_* recovery in cultured astrocytes (Fig. 2*F*).

##### V-ATPase Is the Principal Acid Extrusion Mechanisms in Optic Nerve Astrocytes

**FIGURE 3.**
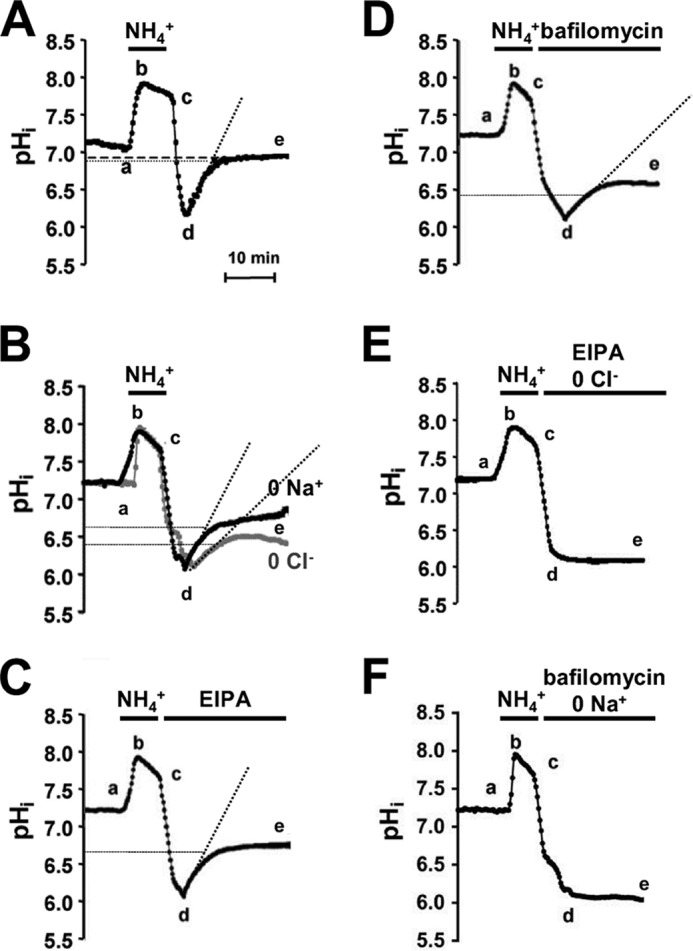
**HCO_3_^−^-independent H^+^ extrusion in neonatal optic nerve astrocytes.** *A*, mean pH*_i_* changes in HEPES-aCSF following a 5-min 20 mm ammonia pulse (*segment a-c*). pH*_i_* recovered after return to ammonia-free conditions (*segment d-e*), but not completely (note *thick dashed line*). *B*, as in A; the recovery was reduced by removal of extracellular Na^+^ (0 Na^+^) and Cl^−^ (0 Cl^−^). *C* and *D*, mean pH*_i_* recovery following acid load in the presence of the Na-H exchange inhibitor EIPA or the V-ATPase inhibitor bafilomycin. Both inhibitors reduced the recovery, although bafilomycin reduced it to the greatest extent. *E* and *F*, pH*_i_* recovery was prevented by the following combinations: NHE inhibitor EIPA and Cl^−^ removal or V-ATPase inhibitor bafilomycin combined with Na^+^ removal. *Dotted lines* on *A–D* indicate recovery rates within the first 5 min following removal of NH_4_^+^. All values are given as mean ± S.E.

We determined the Na^+^ and Cl^−^ dependence of acid extrusion in optic nerve astrocytes, using ammonia pulse recovery. Baseline pH*_i_* was 7.14 ± 0.08 (*n* = 4 optic nerves, 60 cells), and following the acid load, pH*_i_* did not fully recover, returning to a mean of 6.81 ± 0.07 (*p* < 0.001, Fig. 3*A*). The recovery from an acid load in Cl^−^-free medium was significantly reduced in optic nerve astrocytes, reaching a plateau pH*_i_* of 6.36 ± 0.16 (*p* < 0.001 *versus* control), whereas pH*_i_* recovered to 6.78 ± 0.12 in Na^+^-free medium (*p* < 0.001 *versus* Cl^−^-free; *n* = 4 nerves, 60 cells, Fig. 3*B*) after 20 min. Following ammonia loading, perfusion with the NHE inhibitor EIPA resulted in pH*_i_* recovery to 6.76 ± 0.10 (*n* = 4 nerves, 60 cells; Fig. 3*C*), comparable with the recovery in Na^+^-free medium (*p* > 0.89). In the presence of the V-ATPase inhibitor bafilomycin, pH*_i_* recovery reached a steady value of 6.57 ± 0.13 (Fig. 3*D*, *n* = 4 optic nerves, 60 cells), not significantly different from that obtained in Cl^−^-free medium (*p* > 0.31). A combination of EIPA + Cl^−^-free medium (Fig. 3*E*) or bafilomycin + Na^+^-free medium (Fig. 3*F*) prevented pH*_i_* recovery following an acid load.

pH*_i_* recovery rates calculated for the 5-min period following the removal of ammonia confirm the significance of V-ATP in optic nerve astrocytes. The rate in control HCO_3_^−^-free conditions was 0.15 pH units/min, whereas recovery in the absence of NHE (0 Na^+^ or EIPA) or V-ATPase activity (0 Cl^−^ or bafilomycin) was 0.12 and 0.07 pH units/min, respectively. These results demonstrate that V-ATPase, in addition to maintaining resting pH*_i_*, is the major contributor to acid extrusion following an acidic challenge in optic nerve astrocytes.

**FIGURE 4.**
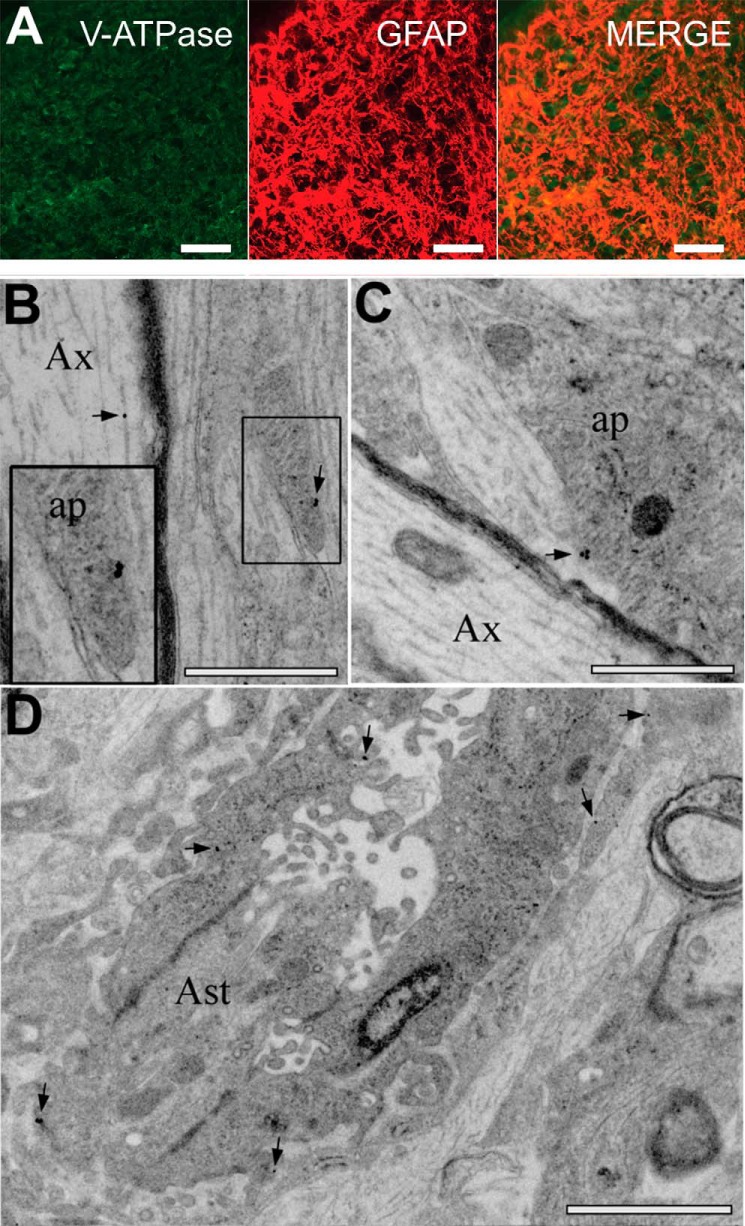
**V-ATPase expression in P2 rat optic nerve.** *A*, V-ATPase (*green*, *left*), and GFAP (*middle*, *red*) expression in the same section, overlaid on the *right*. Note the clear astrocytic expression of V-ATPase, in addition to expression between astrocytes that may correspond to axonal protein. *B–D*, immuno-gold labeling of electron micrographs for V-ATPase reactivity in the long section, showing gold particles (*arrows*) localized to the membrane of astrocyte processes (*ap*, *B* and *C*), and somata (*Ast*, *D*). Note that axons (*Ax*) also contain particles. Note the presence of glial filaments in the astrocyte processes and the wide-bore endoplasmic reticulum, dark bodies, and glycogen particles in the somata (see Ref. 17). The *box* in *B* is shown at higher magnification. *Scale bars* = 10 μm in A; 500 nm in *B* and *C*; and 1 μm in *D*.

Immuno-staining showed co-expression of V-ATPase and the astrocyte marker GFAP, confirming the presence of significant V-ATPase expression in neonatal optic nerve astrocytes (Fig. 4*A*). At the ultrastructural level, immuno-gold labeling of V-ATPase revealed that the protein was present in the cell membrane of astrocyte processes neighboring axons (Fig. 4, *B* and *C*) and in astrocyte somata (Fig. 4*D*), in addition to the previously documented staining of axonal vesicular tubular complex (18). The contribution of V-ATPase to pH*_i_* regulation in these cells was therefore not a result of H^+^ loading into glial synaptic-type vesicles.

##### Buffering Capacity and Extracellular pH

**FIGURE 5.**
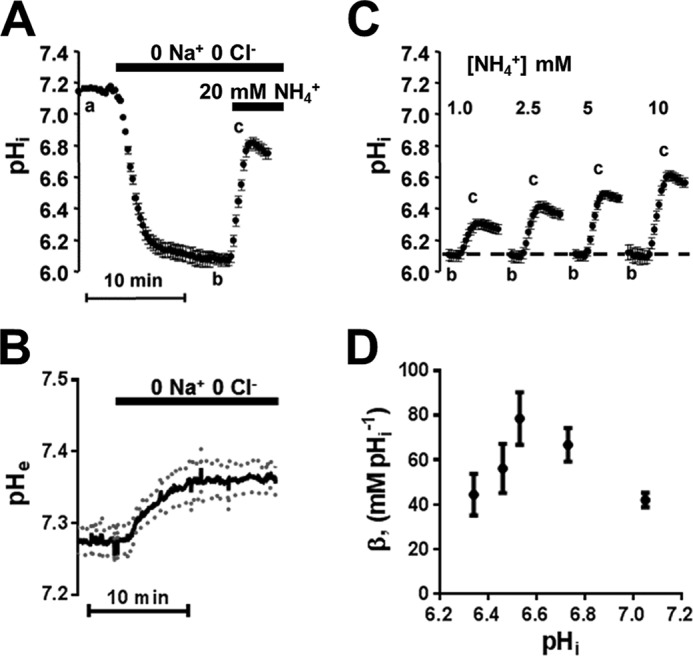
**Intrinsic buffering capacity and pH*_o_* were determined following blockade of Na-H exchange and V-ATPase acid extrusion by NMDG gluconate substitution of Na^+^ and Cl^−^.** *A*, Na^+^ and Cl^−^ removal produced a 1.08 ± 0.08 pH units acidification in optic nerve astrocytes, and exposure to NH_4_^+^ (20 mm) in the absence of acid extrusion mechanisms evoked an alkalinization. *B*, pH*_o_* measured in optic nerve using ion-sensitive microelectrodes was increased by 0.08 pH units following Na^+^ and Cl^−^ removal. *C* and *D*, exposure to NH_4_^+^ as in *A* (1–10 mm) resulted in increasing alkalinization. The buffering capacity (β_i_) for each [NH_4_^+^] concentration was calculated and plotted against the resulting pH*_i_*. Note that the pH change has been extrapolated back to time 0 for each solution. All values are given as mean ± S.E.

The buffering capacity of astrocyte *in situ* has never been assessed, and calculation of the buffering capacity requires displacement of pH*_i_* by known acid loads in the absence of active pH regulation. This is generally achieved by exposure to various NH_4_^+^ concentrations, and the calculation hereof requires knowledge of both pH*_i_* and extracellular pH (pH*_o_*). pH*_i_* regulation was eliminated in optic nerve astrocytes by removal of Na^+^ and Cl^−^, producing a decrease in pH*_i_* from the resting point of 7.15 to 6.07 ± 0.02 (*p* < 0.001; *n* = 4 nerves, 60 cells, Fig. 5*A*). pH*_o_* in neonatal rat optic nerve superfused with HEPES-buffered aCSF (pH 7.45) deviated from that of the bath due to the presence of diffusion barriers between the two compartments and was recorded as 7.28 ± 0.02 (*n* = 4 nerves) using ion-sensitive microelectrodes (Fig. 5*B*). Perfusion with 0 Na^+^/0 Cl^−^ produced a small alkaline increase in the nerve extracellular space to pH 7.36 ± 0.02 (*n* = 4, *p* < 0.05, Fig. 5*B*). Following interruption of pH*_i_* regulation by the ionic substitution, optic nerve astrocytes were acid-loaded with NH_4_^+^ at various concentrations (8) (Fig. 5, *A* and *C*), and the intrinsic buffering capacity (β_i_) was calculated (see “Experimental Procedures”). There was a non-linear relation between β_i_ and pH*_i_* with a maxima around pH 6.5.

## DISCUSSION

The ease with which mammalian astrocyte cultures can be generated and the control the preparation allows of the extracellular medium have resulted in a particular focus on the physiological properties of these cells. When it comes to ion homeostasis, cell culture studies have revealed the range of transport proteins that astrocytes can express and the physiological functions that they can perform. The list of pH-regulating membrane proteins identified in this way includes NHE, Na-HCO_3_ cotransport, Na^+^-driven Cl-HCO_3_ exchange, Cl-HCO_3_ exchange, and a V-ATPase, most of which have also been functionally identified in lower animal preparations (see Refs. 2 and 3 for reviews). The few studies of pH regulation in mammalian astrocytes *in vivo* or in whole-mount preparations indicate the presence of Na-HCO_3_ cotransport in adult retinal astrocytes (19) and of Na-HCO_3_ cotransport and NHE in P8–20 medullary astrocytes (20). The current investigation is the first to examine the contribution made by the transporters NHE and V-ATPase in the nominal absence of HCO_3_^−^ in a whole-mount astrocyte population, revealing the predominance of V-ATPase, a finding with significant functional implications for CNS pH handling.

### 

#### 

##### Neonatal White Matter Astrocytes

The white matter astrocyte population in the CNS matures early relative to other neural cell types. Arising from progenitor cells, including radial glia (21, 22), mature phenotype GFAP^+^ astrocytes are present in the human fetal brain from about post-conception weeks 11 to 14 (23), and populate the intermediate zone that will ultimately form cortical white matter from post-conception weeks 16 to 18 (24). Fibrous white matter astrocytes are established particularly early; for example, they appear in the cortex before protoplasmic gray matter astrocytes (24, 25). Studies of astrocyte development in central white matter are complicated by the complex morphological arrangement of axon tracts at different maturational stages. Large differences in astrocyte development have been noted, for example, between closely apposed fiber tracts in the optic radiations (24). These complications are avoided in the optic nerve, which is a uniform and structurally isolated white matter tract where astrocytes are generated from astrocyte precursor cells rather than by transformation of radial glia (26–29). By mid-gestation in humans, optic nerve astrocyte maturation is largely complete, predating the onset of myelination by ∼20 weeks (26).

In animal studies, astrocyte precursor cells populate the RON at fetal day 17, and GFAP^+^ astrocytes are generated continually until the end of the first week of life (27–29). At birth, >70% of cells in the RON are astrocytes with many of the features of mature cells including a radiating stellate morphology, expression of glial filaments, and organelle structures typical of the mature phenotype (such as endoplasmic reticulum, mitochondria, and Golgi). These astrocytes selectively accumulate AM-conjugated dyes (12), and the current study has taken advantage of the early maturation of fibrous astrocytes to examine pH*_i_* regulation in an effectively intact whole-mount preparation.

##### Astrocytes in Situ Rely Primarily on V-ATPase for HCO_3_^−^-independent pH_i_ Regulation

V-ATPase is thought to be present in nearly every eukaryotic cell, where it acidifies intracellular organelles such as lysosomes, secretory, and synaptic vesicles. In the plasma membrane, V-ATPase functions to acidify the extracellular space, facilitating nephronal acidification, resorption of bone by osteoclasts, or reabsorption of bicarbonate in renal proximal tubules (reviewed in Refs. 32 and 33). In the absence of HCO_3_^−^, NHE1 has been identified as the principal acid extrusion mechanism in astrocytes (10, 30, 31) (see Ref. 3 for review), with V-ATPase being a minor contributor in cultured rat hippocampal astrocytes (10) and not involved in baseline pH*_i_* maintenance in cultured cortical astrocytes (30). V-ATPase is dependent upon extracellular [Cl^−^] (34–36), and consistent with the low activity of V-ATPase in cultures, we found only a minor change in pH*_i_* of cultured astrocytes upon Cl^−^ removal or block by the selective inhibitor bafilomycin. In optic nerve astrocytes, these conditions resulted in significant acidification, leading to a pH*_i_* convergence in the two astrocyte populations. HCO_3_^−^-independent pH recovery in cultured astrocytes was unaffected by Cl^−^ removal, but was highly Na^+^-dependent. These findings align with data on astrocyte cultures showing that H^+^ extrusion in HEPES-buffered medium at a pH*_i_* of 6.05 is mediated by EIPA- and amiloride-sensitive NHE (8). In comparison, pH recovery from an acid load in optic nerve astrocytes exhibited little Na^+^ or EIPA sensitivity, whereas Cl^−^ removal or bafilomycin greatly reduced acid extrusion. These data are consistent with the hypothesis that HCO_3_^−^-independent pH*_i_* regulation in optic nerve astrocytes is mediated predominantly by V-ATPase with NHE playing a minor role. Consistent with this hypothesis, immuno-staining at the light and ultrastructural levels shows robust V-ATPase expression in the cell membrane of these astrocytes.

##### Buffering Capacity of Astrocytes in Situ

Buffering power in cultured cerebral and hippocampal astrocytes is essentially constant in the pH range 6–7 (37, 38) or linearly decreases with increasing pH*_i_* (8, 31). We found a non-linear relation between buffering power and pH*_i_* in optic nerve astrocytes. Measurements of buffering power are inherently variable, especially at low pH values (*e.g.* Ref. 31), and any inferred linear relationship with pH is possibly unwarranted. Buffering power distribution is the sum of the concentration and p*K_a_* of all proton-buffering molecules in the cell (39); the buffering distribution therefore depends on the cellular composition and will not necessarily follow pH linearly.

We found that the intrinsic buffering capacity of the optic nerve astrocytes was above 40 mm (pH)^−1^ throughout the pH range ∼6.3–7.1. This is in the 26–50 mm (pH)^−1^ range found in hippocampal slices, whole-brain cortical tissue homogenate, and isolated synaptosomes (37, 40–42), but substantially greater than the 10.5–14.1 mm (pH)^−1^ range of cultured astrocytes (8, 31, 38). It is possible that buffering capacity is generally reduced in cultured cells as compared with cells *in vivo*. A high intrinsic buffering capacity paired with a presumably higher bicarbonate buffering power due to the elevated resting pH*_i_* of *in situ* astrocytes would render the cells more resistant to pH fluctuations than their cultured counterparts.

##### Conclusion

Excitability in the neonatal rat optic nerve is mediated by long-duration, slow-conducting action potentials (43), and excitability-dependent changes in the extracellular space are exaggerated in this tissue. Extracellular [K^+^] can rise ∼20 mm during electrical stimulation of neonatal optic nerve axons (44), and neighboring astrocytes express relatively high levels of voltage-gated Na^+^ channels, which are down-regulated in the absence of axonal contact (45). Large activity-dependent elevations in extracellular [K^+^] are likely to evoke significant rises in intracellular [Na^+^] in these astrocytes, which would influence pH*_i_* regulation mediated via NHE. This astrocyte population may therefore require a form of pH*_i_* regulation that is independent of intracellular [Na^+^], such as that provided by V-ATPase. Reliance upon V-ATPase for HCO_3_^−^-independent pH*_i_* regulation, in contrast to NHE, may therefore have functional utility for neonatal white matter astrocytes. However, the assumption that CNS astrocytes in general express low levels of V-ATPase and high levels of NHE is based almost entirely upon cell culture studies, and the current findings question the validity of this assumption. Voltage-gated Na^+^ channels and Na^+^-dependent transport expression are widespread among astrocyte populations, and intracellular [Na^+^] has recently been implicated in the regulation of astrocyte signaling at synapses (46). Synapses are also sites where pH*_i_* regulation is an essential function of astrocyte processes; for example, activity-dependent acidification at NMDA-type glutamate receptors would have profound consequences for synaptic integration (47). Na^+^-independent pH*_i_* regulation may therefore be a general requirement for perisynaptic astrocyte processes. Because astrocytes *in vitro* do not form processes that are integrated into a neural network, they may fail to express the levels of V-ATPase found in the current study.

In general, astrocytes expressing high levels of V-ATPase will have an elevated capacity for pH*_i_* regulation, in particular at the acid pH levels that prevail under ischemic conditions in the CNS. These are conditions under which such a transporter might be required because HCO_3_^−^ levels will be reduced, limiting HCO_3_^−^-dependent pH*_i_* regulation, whereas NHE is unlikely to function efficiently due to reduced extracellular pH (3). In this regard, it is interesting that astrocytes contain the only significant energy reserve in the CNS in the form of glycogen (48), and V-ATPase may therefore participate in pH maintenance during limited periods of ischemia. High levels of functional V-ATPase expression in astrocytes therefore may have important implications for both the physiology and the pathophysiology of the CNS.

## Footnotes

2 The abbreviations used are:

NHE: Na-H exchanger
BCECF: 2′,7′-bis-(2-carboxyethyl)-5 (and 6)-carboxyfluorescein
AM: acetoxymethyl
aCSF: artificial cerebrospinal fluid
GFAP: glial fibrillary acidic protein
NMDG: *N*-methyl-d-glucamine
EIPA: ethyl-isopropyl amiloride
RON: rat optic nerve
P: postnatal day(s).
